# Local biochemical and morphological differences in human Achilles tendinopathy: a case control study

**DOI:** 10.1186/1471-2474-13-53

**Published:** 2012-04-05

**Authors:** Pingel J, Fredberg U, Qvortrup K, Larsen JO, Schjerling P, Heinemeier K, Kjaer M, Langberg H

**Affiliations:** 1 Institute of Sports Medicine, Department of Orthopedic Surgery M. Bispebjerg Hospital and Center for Healthy Aging, Faculty of Health Sciences, University of Copenhagen, Copenhagen, Denmark; 2 Department of Rheumatology, Silkeborg Hospital, Silkeborg, Denmark; 3 Department of Biomedical Sciences, University of Copenhagen, Copenhagen, Denmark; 4 Department of Neuroscience and Pharmacology, Faculty of Health Sciences, University of Copenhagen, Copenhagen, Denmark; 5 Department of Public Health, University of Copenhagen, Copenhagen, Denmark

**Keywords:** Collagen, Gene expression, Patients, Growth factors, Tissue turnover

## Abstract

**Background:**

The incidence of Achilles tendinopathy is high and underlying etiology as well as biochemical and morphological pathology associated with the disease is largely unknown. The aim of the present study was to describe biochemical and morphological differences in chronic Achilles tendinopathy. The expressions of growth factors, inflammatory mediators and tendon morphology were determined in both chronically diseased and healthy tendon parts.

**Methods:**

Thirty Achilles tendinopathy patients were randomized to an expression-study (*n *= 16) or a structural-study (*n *= 14). Biopsies from two areas in the Achilles tendon were taken and structural parameters: fibril density, fibril size, volume fraction of cells and the nucleus/cytoplasm ratio of cells were determined. Further gene expressions of various genes were analyzed.

**Results:**

Significantly smaller collagen fibrils and a higher volume fraction of cells were observed in the tendinopathic region of the tendon. Markers for collagen and its synthesis collagen 1, collagen 3, fibronectin, tenascin-c, transforming growth factor-β fibromodulin, and markers of collagen breakdown matrix metalloproteinase-2, matrix metalloproteinase-9 and metallopeptidase inhibitor-2 were significantly increased in the tendinopathic region. No altered expressions of markers for fibrillogenesis, inflammation or wound healing were observed.

**Conclusion:**

The present study indicates that an increased expression of factors stimulating the turnover of connective tissue is present in the diseased part of tendinopathic tendons, associated with an increased number of cells in the injured area as well as an increased number of smaller and thinner fibrils in the diseased tendon region. As no fibrillogenesis, inflammation or wound healing could be detected, the present data supports the notion that tendinopathy is an ongoing degenerative process.

**Trial registration:**

Current Controlled Trials ISRCTN20896880

## Background

Tendons connect muscle to bone and enable transmission of forces from contracting muscle to bone, resulting in joint movement. They possess the ability to adapt to changes in loading [1] and studies have shown that collagen synthesis is increased as a result of both acute exercise [2,3] and prolonged physical training [4]. The adaptation to loading can ultimately lead to increases in CSA and collagen content in chronically loaded tendons [5]. Despite this physiological ability to adapt, tendinopatic tendons represents a large and constantly growing clinical problem affecting both recreational and professional athletes as well as people involved in repetitive labour [6,7]. Years of research have unfortunately not provided much insight into the pathogenesis of chronic tendinopathy [8]. Indeed, the etiology of tendinopathy has been related to repeated micro strain below the failure threshold as an initiating stimulus for degenerative processes [9,10]. Other authors, however, have proposed that mechano-biological under-stimulation results in a degenerative cascade, through the production of a pattern of catabolic gene expression that leads ultimately to extracellular matrix degeneration [11]. Tendinopathy is characterized by activity-related pain, focal tendon tenderness, and decreased local movement in the affected area [12,13]. The general opinion is that no inflammatory cells are present in the tendinopathic tissue [14] and that tendinopathy is the result of a degenerative process with collagen disorganization, collagen fibre separation, increased cellularity, neovascularization and focal necrosis [15].

Previous studies have shown an altered concentration of certain matrix metalloproteinases MMPs, AdAMt's and TIMP's in normal and degenerate human Achilles tendon [16]. Additionally several cytokines [9,10] can be found in tendons and fibroblasts after cyclic mechanical stretching in healthy tendon tissue. However, the published data arises from the comparison of tendinopathic tissue with either control tissue from different anatomical tendons [17] or with tissues from identical anatomical tendons but from different subjects [18]. Since considerable microscopic structure differences have been demonstrated in anatomically different tendons [19], this limits the conclusions that may be drawn from these studies. Taking the aforementioned limitations into account, current data concerning local biochemical differences within tendinopathic tendons, seem to indicate that an altered expression of collagen [20], proteoglycans [21] and matrix metalloproteinases [16,22] exists in tendinopathic tendons. In addition the level of cytokines [23] VEGF and fibronectin [24] has been shown to be significantly different in the tendinopathic area. However analyses of local biochemical differences together with morphological differences are lacking.

The aim of the present study was to elucidate if any local structural differences are present in tendinopathic areas of human Achilles tendons compared to healthy areas in the same tendon. Furthermore, we wanted to investigate which proteoglycans, growth factors and cytokines that were involved in the local structural differences observed.

We hypothesize that several markers such as collagen 3 would be locally up regulated indicating formation of scar tissue with in the tendon [25] and higher concentrations of MMP-2 and MMP-9 indicating an enhanced degradation of collagen structures in the tendinopathic area (t-area) when compared with the healthy area (h-area) of the same tendon. Furthermore it is hypothesized that certain proteoglycans would have altered expression in the two tendon regions, e.g. an increased expression of decorin which might cause the collagen turnover to be increased also in chronic tendinopathic tendons. Additionally we hypothesize that growth factors like fibroblast growth factor (bFGF) are decreased causing a reduced healing capacity in the injured area of the Achilles tendons.

## Methods

### Design

Thirty patients with chronic Achilles tendon pain were included in this study approved by the local Ethical Committee of the Capital Region Copenhagen (H-1-2009-114) and in compliance with the Helsinki Declaration. Additionally the study was registered at Current Controlled Trials (ISRCTN20896880). Due to limitations in the amount of tissue gained from the tendon biopsies patients were randomly assigned to either a Structural study (*n *= 14) or a Biochemical study (*n *= 16) by the envelope method. All subjects were recreational athletes or workers with a long-term history of chronic Achilles tendon pain (> 1/2 year) (Table 4) and conventional conservative treatments (eccentric rehabilitation, NSAIDs and corticosteroid injections) had been tried in all individuals with no effect. Intake of NSAID or corticosteroid injection was not allowed 6 months prior to inclusion in the present study. All subjects were recruited from the Rheumatology Department, Silkeborg Hospital, Denmark, and the biopsies from the Achilles tendons were taken as part of a standard procedure in order to examine for deposits of cholesterol, uric acid, and amyloid in the injured Achilles tendons.

### Biopsy procedure

The subjects were locally anesthetized, in the peritendinous space from both the medial and lateral side of the tendon with injections of 2 × 10 ml 1% Lidocain, using ultrasound guidance. Biopsies were taken with a semi-automatic biopsy needle (14 GA, 9 cm; Angiotech) also using ultrasound (US) guidance. An initial tendon biopsy was taken in the maximally sick area evaluated using US (defined as the area with maximal increased tendon thickness, neovascularisation, hypoeccogenicity). This area was usually 3-5 cm above the attachment of the Achilles tendon to the calcanaeus bone. A second biopsy was taken from the same tendon 4 cm proximal to the first biopsy in a region of the tendon tissue that was deemed normal using US.

Biopsy samples intended for analysis using Transmission Electron Microscopy were immersed in 2% glutaraldehyde in 0.05 M sodium buffer (pH 7.2), and the samples for gene expression were snap-frozen and stored at -80°C until analysis.

### Transmission electron microscopy of tendon biopsies

Fourteen tendon biopsy pairs were cut into small pieces and were immersion-fixed in 2% glutaraldehyde for 24 hours. Following three rinses in 0.15 M sodium phosphate buffer (pH 7.2) the specimens were post-fixed in 1% OsO_4 _in 0.12 M sodium cacodylic buffer for 2 hours. The specimens were dehydrated in a graded series of ethanol (70%, 96% and 100%), transferred to propylene oxide and embedded in Epon (VWR Bie&Berntsen) in three steps according to standard procedures. For each biopsy one ultra thin section was cut approximately perpendicular to the length axis of the tendon with a Reichert-Jung ultracut E microtome. The section was collected on a one-hole copper grid with a Formvar supporting membrane and stained with uranyl acetate and lead citrate. The sections were examined using a Phillips CM 100 transmission electron microscope operated at an accelerating voltage of 80 kV. Digital images were obtained with a MegaView II camera and an analysis software package. From each ultra thin section the intercellular tissue was examined by taking a simple, random sample of ten digitized TEM images of the intercellular tissue. The cellular component of the tendon was examined in eleven biopsy pairs by taking 6 times 6 images in three randomly positioned regions of the section. The 6 times 6 images were spliced into one image using multiple image alignment (MIA) tools, so for each examined biopsy a total of three MIA images were obtained.

### Stereology

The Stereological analyses of the images were carried out on a computer monitor onto which the digitized EM image was merged with a graphic representation of the stereological test systems for just 12 of the 14 biopsy pairs (2 biopsies was unfortunately not useable for stereology analyses) (C.A.S.T.-grid software, The International Stereology Center at Olympus). The intercellular tissue was analyzed at a final magnification of 210.000 in the ten ordinary TEM images. The volume fraction (Vv) of collagen fibrils per intercellular tissue volume was estimated with the point counting technique as the number of points hitting collagen fibres divided by the number of points hitting the intercellular tissue (including collagen fibrils) using a point grid of 36 points. The number of collagen fibrils per cross sectional area of intercellular tissue (NA) was counted in 16 uniformly positioned, unbiased counting frames, each with an area of 0.0426 mm^2 ^(42.6 μm^2^), and the individual diameters (d) of the sampled collagen fibrils were measured as the largest diameter perpendicular to the longest axis (i.e. the length of the minor axis of the ellipse) using the "measure-length" feature of the CAST-grid system. The unbiased counting frame ensures that all profiles, regardless of shape, size or orientation, have an equal probability of being sampled within area probe. The MIA images were analyzed at a final magnification of 115,000. The point counting technique, using a point grid with approximately 1000 points, estimated the volume fractions of the cellular component of the tendon tissue. The estimated parameters were: the volume fraction of cells within the tendon, the volume fraction of the nucleus within the cell, and the volume fraction of cytoplasm within the cell. A single experienced investigator performed all stereological analyses in a blinded fashion. The investigator was blinded for all subject characteristics, and whether the sample was obtained from the tendinopathic or the healthy region of the tendon.

### RNA extraction and real time-PCR analysis

Total RNA isolation: Total RNA was extracted from frozen tendon samples from 16 subjects (sample weight: mean 23.2 ± 6.4 mg) by using 1 ml of TRI Reagent (Molecular Research Centre, Cincinnati, OH) 5 steel beads (2.3 mm) and 4 silica beads (1.0 mm Silicon Carbide Beads (454 grams) BioSpec Products Inc.). Glycogen was added (120 μg per ml of TriReagent) to the tendon samples to improve RNA precipitation.

Extracted RNA was precipitated from the aqueous phase with isopropanol and was washed with ethanol [75%], dried and suspended in 10 μl of nuclease-free water. The RNA concentration was determined using a RiboGreen RNA Quantitation kit 200-2000 Assays, Molecular Probes USA. RNA quality was determined on the basis of a RNA 6000 nano Chip assay kit, Agilent Technologies, Germany. The RNA samples were stored frozen at -20°C until subsequent use in real-time RT-PCR procedures.

cDNA synthesis: 100 ng RNA was reverse transcribed for each tendon sample in a total volume of 20 μl by using the QiagenOmniscript RT Kit at 37°C for 1 hour followed by 70°C for 15 minutes. The resulting cDNA was diluted twenty times in dilution buffer (10 mMTris EDTA buffer: Sigma Germany) + Salmon Testes DNA (1 ng/μl; Sigma Germany), and samples were stored at -20°C until used in the PCR reactions for specific mRNA analysis.

Polymerase Chain Reaction: The Real-time PCR-method using Glyceraldehyde 3-phosphate dehydrogenase (GAPDH) and 60S acidic ribosomal protein P0 (RPLP0) as reference genes to study specific mRNA's of interest was applied. The primers were purchased from MWG Biotech. For each target cDNA the PCR reactions were carried out under identical conditions by using 5 μl diluted cDNA in a total volume of 25 μlQuantiTect SYBR Green PCR Mix (Qiagen) and 100 nM of each primer (Table 2). The amplification was monitored in real-time using a MX3005P real-time PCR machine (Stratagene, CA). The threshold cycle (C_t_) values were related to a standard curve made with cloned PCR products to determine the relative difference between the unknown samples, accounting for the PCR efficiency. The specificity of the PCR reaction was confirmed by melting curve analysis after amplification. The real-time PCR conditions were as follows: to denaturate the DNA strands the reaction mix was heated above the melting temperature of DNA (95°C) for 10 minutes, followed by 50 cycles each of 15 seconds at 95°C, followed by the annealing step where optimal primer hybridization conditions were obtained by lowering the temperature to 58°C for 30 seconds, and the extension step, where the reaction mix was heated to 63°C for 90 seconds. Two housekeeping genes GAPDH and RPLP0 were used as reference genes. The RPLP0 gene had been chosen as an internal control, assuming RPLP0 to be constitutively expressed. To validate this assumption GAPDH mRNA was measured as another unrelated "constitutive" and normalized with RPLP0, showing no difference between the healthy and the tendinopathic region of the tendon (Figure 3).

### Statistical analysis

The PCR data were log transformed and a Paired Students *t*-test was performed to compare the results from the healthy area of the tendon with the tendinopathic area of the tendon, with exception of the results from IL-6, IL-1b, ki67 and HGF-1. These gene targets could not be detected in all samples. In these cases Chi^2 ^tests were performed. All PCR data are presented as the geo mean ± backtransformed SEM. The collagen fibril data were divided into area and diameter fractions, and a paired Students *t*-test was performed to compare each fraction between the healthy and the tendinopathic area of the tendon. Likewise, the volume fraction of cells and the volume fraction of the nucleus within the cell were compared using a Paired Student *t*-test comparing the two areas of the tendon. A P-value < 0.05 was considered to be significant and all data despite of the subject characteristics are shown as Mean ± SEM.

## Results

### Structural composition of the tendon

**Figure 1:**
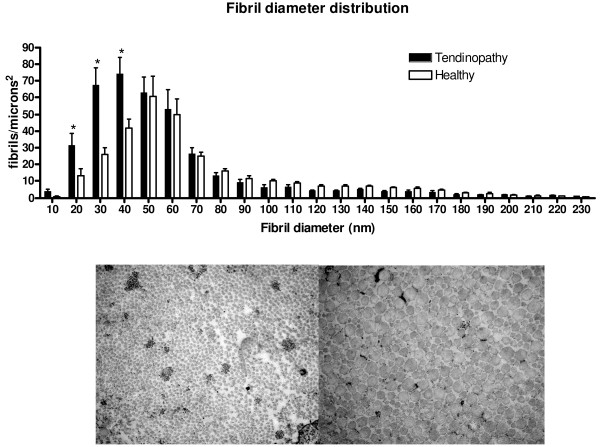
**Fibril diameter distribution**. Fibril diameter of the tendinopathic and the healthy area of the same tendon divided into fractions. Error bars represent SEM (*P *< 0.05).

**Figure 2:**
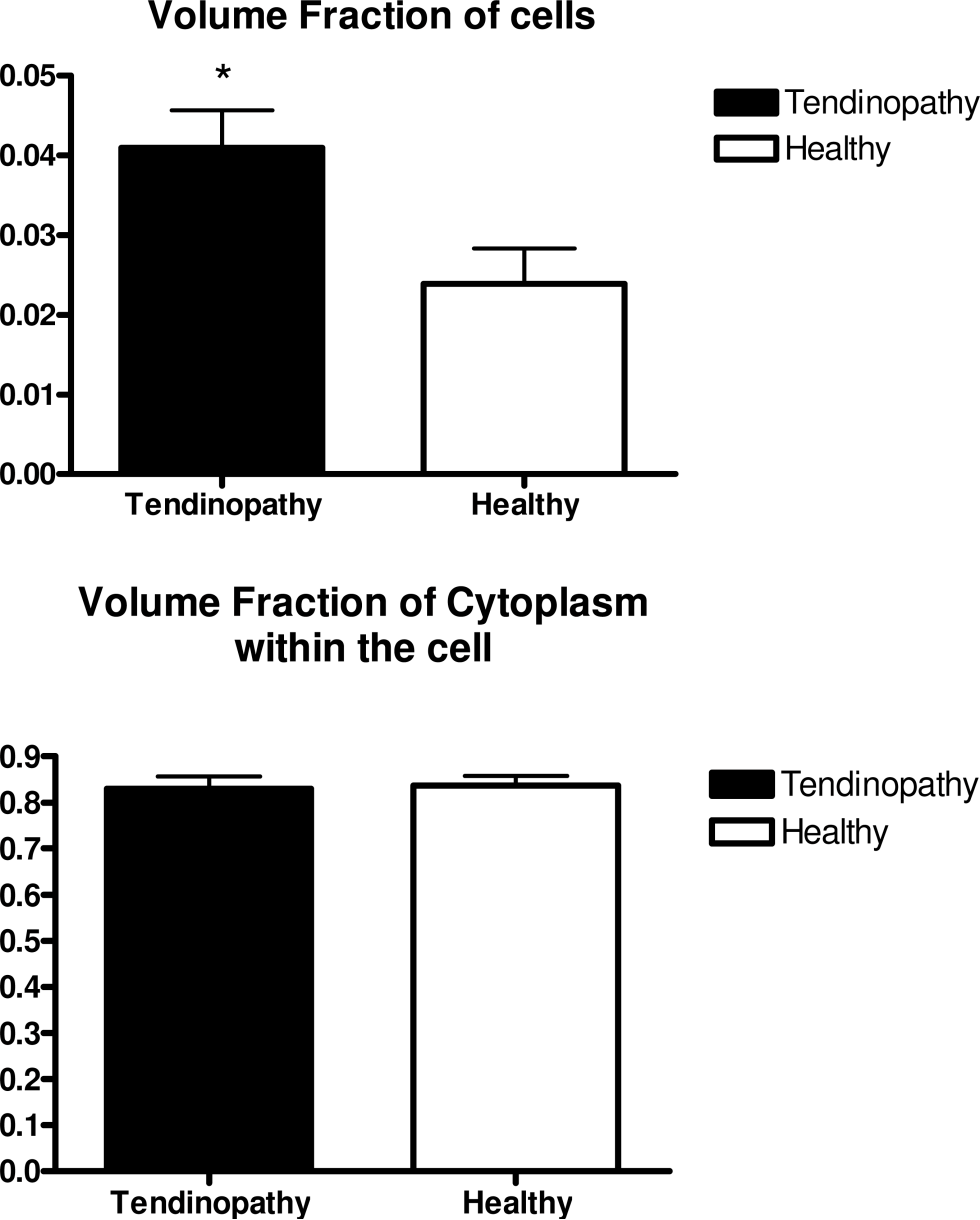
**Volume fraction of cells and cytoplasm within the cells**. Error bars represent SEM. (*P *< 0.05).

The density, volume fraction and mean area of the collagen fibrils were measured in biopsies from 14 of the tendinopathy patients (Table 1). The density of collagen fibrils was found to be significantly higher and the mean area of the collagen fibrils was significantly smaller in tendinopathic tendon region compared to that of healthy control region, additionally a trend towards significant difference was found in volume fraction (Table 1). When analysing the individual bins in the diameter distribution of the fibrils, a significantly higher number of fibrils with a diameter in the lower range (10-40 nm) was found in the tendinopathic area compared to the healthy area (diameter 10-20 nm: Tendinopathic area: 32 ± 7 fibrils/μm^2^; Healthy area 13 ± 4 fibrils/μm^2^; diameter: 20-30 nm: Tendinopathic area: 68 ± 10 fibrils/μm^2^; Healthy area: 26 ± 4 fibrils/μm^2^; diameter 30-40 nm: Tendinopathic area: 74 ± 10 fibrils/μm^2^; Healthy area: 42 ± 5 fibrils/μm^2^; see Figure 1). In addition a significantly higher volume fraction of cells was observed in the tendinopathic area of the tendon (Figure 2). The volume fraction of the cytoplasm within the cell was found to be identical in the two areas (Figure 2) implying an increased number of cells in the tendinopathic area.

**Table 1:** Tendon fibril characteristics

	Sick Tendon Tissue		Healthy Tendon Tissue		
	**Mean**	**SD**	**Mean**	**SD**	***P*-value**

Density	155,73	48,80	111,09	46,72	0,04

Volume Fraction	0,56	0,08	0,61	0,08	0,06

Mean Area	2963,54	1693,29	5239,15	2298,83	0,02

### Gene expression analysis

The gene expression of 24 genes was analyzed in the biopsies of 16 tendinopathy patients (Table 2). As mentioned in the methods section, GAPDH mRNA served as a normalization factor for the genes of interest, and RPLP0 mRNA was used to validate the stability of the expression of GAPDH mRNA. Specific gene targets were selected covering the areas of: Extracellular matrix (ECM) components, degradation components, Growth factors, inflammatory markers and fibrillogenesis.

**Table 2:** PCR primers

mRNA	Sense Primer	Antisense Primer
Collagen 1	GGCAACAGCCGCTTCACCTAC	GCGGGAGGACTTGGTGGTTTT

Collagen 3	CACGGAAACACTGGTGGACAGATT	ATGCCAGCTGCACATCAAGGAC

Fibronectin	TTTGCTCCTGCACATGCTTT	TAGTGCCTTCGGGACTGGGTTC

Tenascin C	CAACCATCACTGCCAAGTTCACAA	GGGGGTCGCCAGGTAAGGAG

Fibromodulin	CAGTCAACACCAACCTGGAGAACC	TGCAGAAGCTGCTGATGGAGAA

Versican	AGTCAGTGGAAGGCACGGCAATCT	CCGTTAAGGCACGGGTTCATTT

Decorin	GGTGGGCTGGCAGAGCATAAGT	TGTCCAGGTGGGCAGAAGTCA

MMP-2	CCGCCTTTAACTGGAGCAAAAACA	TTGGGGAAGCCAGGATCCATTT

MMP-9	AGCGAGGTGGACCGGATGTT	AGAAGCGGTCCTGGCAGAAATAG

TIMP-1	CGGGGCTTCACCAAGACCTACA	TGGTCCGTCCACAAGCAATGA

TIMP-2	CTCGCTGGACGTTGGAGGAAAG	GTGTCCCAGGGCACGATGAAGT

CTGF	TGCGAAGCTGACCTGGAAGAGA	GCCGTCGGTACATACTCCACAGAA

bFGF	TGACGGGGTCCGGGAGAAGA	ATAGCCAGGTAACGGTTAGCACACAC

HGF	TGAAATATGTGCTGGGGCTGAAA	ACAAACAAGTGGGCCACCATAATCC

cMet	AACCCGAATACTGCCCAGACCC	TGATATCCGGGACACCAGTTCAG

VEGFA-1	ATGACGAGGGCCTGGAGTGTGT	CTCCTATGTGCTGGCCTTGGTG

IGF-1	GACATGCCCAAGACCCAGAAGGA	CGGTGGCATGTCACTCTTCACTC

TGFb-1	GAGGTCACCCGCGTGCTAATG	CACGGGTTCAGGTACCGCTTCT

Cox-1	GGTTTGGCATGAAACCCTACACCT	CCTCCAACTCTGCTGCCATCT

IL-1R	GGAAGGGATGACTACGTTGGGGA	CCAGCCAGCTGAAGCCTGATGTT

IL-1b	TCCAGGGACAGGATATGGAGCA	AGGCCCAAGGCCACAGGTATTT

KI67	CGGAAGAGCTGAACAGCAACGA	GCGTCTGGAGCGCAGGGATA

CCL	GCCCTTCTGTGCCTGCTGCT	GCAGGTGACTGGGGCATTGATT

IL-6	GAGGCACTGGCAGAAAACAACC	CCTCAAACTCCAAAAGACCAGTGATG

TNF-a	TTCCCCAGGGACCTCTCTCTAATC	GAGGGTTTGCTACAACATGGGCTAC

RPLP0	GGAAACTCTGCATTCTCGCTTCCT	CCAGGACTCGTTTGTACCCGTTG

GAPDH	CCTCCTGCACCACCAACTGCTT	GAGGGGCCATCCACAGTCTTCT

**Figure 3:**
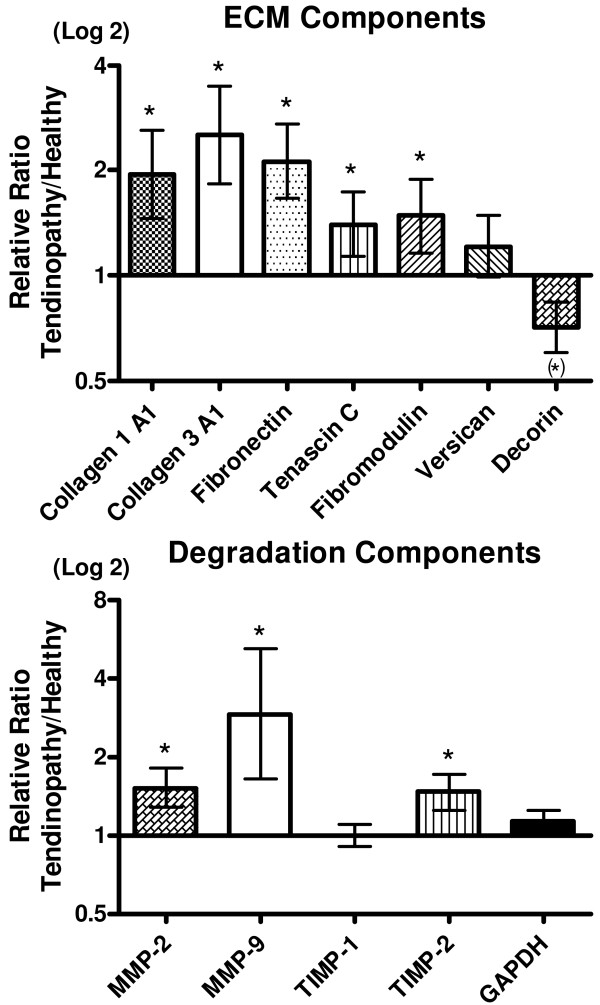
**Collagen turnover**. Gene expressions of collagens, non-collagenous matrix components, matrix metalloproteinases and metallopeptidase inhibitors, shown as a relative ratio between the tendinopathic and the healthy region of the tendon. The healthy region equals 1. Error bars represent SEM. (*P *< 0.05).

Extracellular matrix components: The expression of several structural components (collagen 1 and collagen 3) together with mRNA of fibronectin, tenascin-c and fibromodulin was found to be significantly up regulated in the t-area (Figure 3). Versican was found to be unchanged and decorin tended to be decreased in the t-area compared to that of the h-area of the Achilles tendon (Figure 3).

Degradation factors: Expression of MMP-2, MMP-9 and TIMP-2 was significantly increased in the t-areas compared to that of the h-areas with no difference in expression of TIMP-1 (Figure 3).

**Figure 4:**
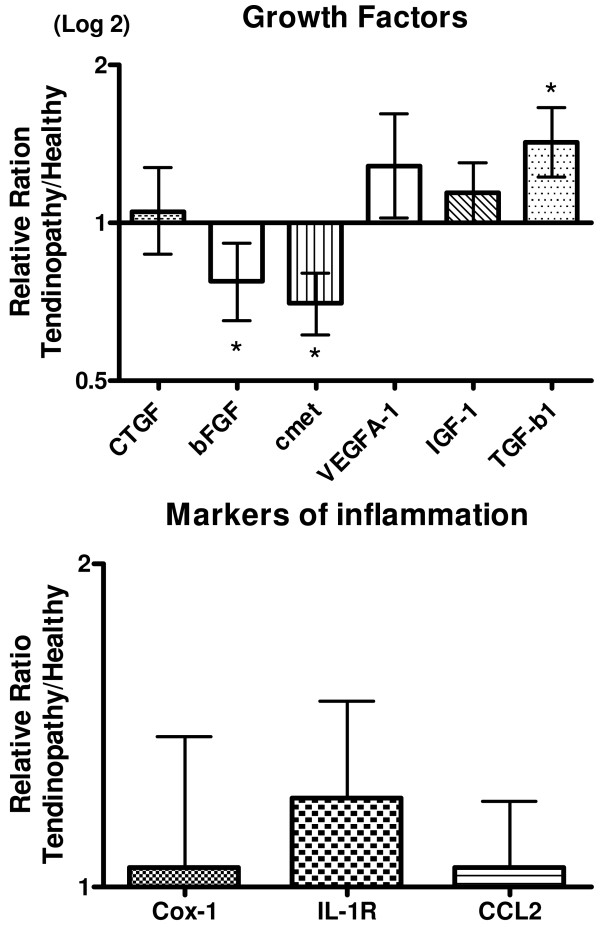
**Tendon healing**. Gene expressions of Growth factors and different inflammatory markers, shown as a relative ratio between the tendinopathic and the healthy region of the tendon. The healthy region equals 1. Error bars represent SEM. (*P *< 0.05).

Growth factors: A significant up-regulation of TGF-β1 expression was observed in the t-area compared to that of the h-area, whereas bFGF and cmet expression in the same area was significantly decreased. No differences were observed in the expression of CTGF, VEGF-A1 and IGF-1 (Figure 4). The expression of HGF-1 could not be detected in all samples but no significant differences were found between the two regions of the tendon (Table 3).

**Table 3:** Inflammatory markers

	Healthy	Tendinopathy	Chi test
	**(detected/not detected)**	**(detected/not detected)**	

IL-6	9/7	10/6	0.7

IL-1b	4/12	6/10	0.4

ki67	10/6	8/8	0.5

HGF-1	10/6	7/9	0.3

total	16	16	

**Figure 5:**
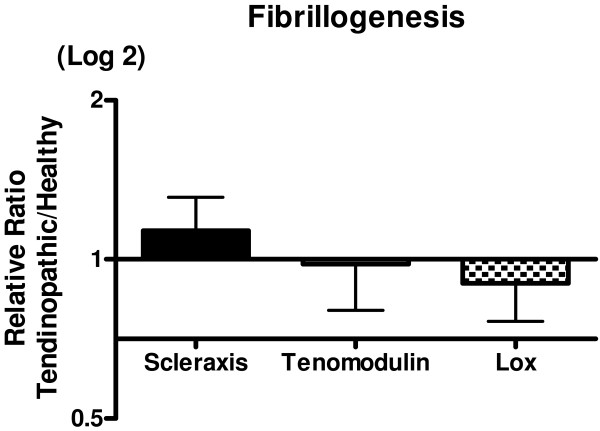
**Fibrillogenesis**. Gene expressions of markers for fibrillogenesis, shown as a relative ratio between the tendinopathic and the healthy region of the tendon. The healthy region equals 1. Error bars represent SEM (*P *< 0.05).

Inflammatory markers: Data on IL-6, IL-1b and ki67 could not be detected in all samples and no differences were observed when the two regions were compared (chi squared test). Ki67 showed a significant decrease in expression in the tendinopathic areas. No expression could be detected of COX-2 and TNF-α in any of the samples (results not shown). COX-1, IL-1R and CCL2 was detected in all samples, but there was no significant difference between the t-area and the h-area of the tendon (Figure 4). Fibrillogenesis: There were no differences in expression of scleraxis, tenomodulin and Lysyloxidase (LOX) between the two areas (Figure 5)

## Discussion

The major finding of the present study is that tendinopathy causes focal biochemical and morphological differences in the human Achilles tendon. The data obtained using TEM indicate that the structural composition of the t-area has a significantly increase in the number of smaller collagen fibrils compared to the h-area of the same tendon. This supports our hypothesis that localized structural differences are present in tendinopathic Achilles tendons, potentially as a result of an increased turnover of the tissue in an attempt to heal potentially injured tissue. This fits with previous findings where a site-specific loss of larger collagen fibrils and an increase of fibrils with a small diameter was observed in Achilles tendons after tendon rupture [26]. However the present findings differ somewhat from the results observed in another study from our laboratory [27], in which a significantly larger fibril area and a lower collagen fibril density was observed in patellar tendinopathy [27]. This discrepancy might partly be explained by the fact of the patella tendon functions more like a ligament (ligament patella) with the primary role of ensuring a fixed distance between the patella apex and the insertion of the tendon on to the tibia bone, in contrast to the Achilles tendon which functions more like a spring, providing a means of shock absorption *via *the connective tissue, during periods of muscle contraction and loading [28]. Since the function of the patellar and the Achilles tendon differ, it is likely that the structure of the tendons e.g. the cross-linking and length of fibrils etc. of the two tendons reflects this difference. A further explanation might be that the healthy tissue was taken from the same tendon in the present study, while Kongsgaard et al. [27] used control tissue taken from another tendon of healthy control subjects. The present design has as all study designs advantages and limitations. The advantage is that this design enables to investigate local differences in the tendon, the limitation is that no control tissue is available from tendons that never had any symptoms. Changes that occur in the whole tendon can therefore be overlooked. A previous study has shown that histological changes in the tendon were not only present at the site of rupture but also in the macroscopical normal part of the tendon, indicating that local alterations of the tissue might not necessarily be local [29]. Whether tendinopathy shows the same pattern is unclear and needs further investigation. However we believe that the present local differences between the t-area and the h-area are strong enough to justify the conclusions of the present study. To investigate if the increased number of small collagen fibrils could be due to genesis of collagen fibrils or degradation of previously much larger fibrils, the gene expression of scleraxis and tenomodulin was analysed. Both gene targets have previously been associated with tendon formation and development [30,31]. The absent difference of the expression of scleraxis and tenomodulin suggests that no fibrillogenesis took place in the t-area at this very late stage in the disease (Figure 5). Furthermore a similar expression of Lysyl oxidase (LOX) in both regions of the tendon indicates that the tissue does not compensate for the localized structural changes by initiating cross-links to maintain the mechanical properties of the tendon. Previously findings showed that training increases the expression of LOX in healthy tendon tissue in rats [32]. The present data suggest that this adaptation does not take place in t-area of the tendon. Several abnormalities of the tendon structure have been investigated with histopathological analysis including fibre structure, fibre arrangement, nuclear rounding and cellularity [15]. In the present study a significant increased volume fraction of cells was observed in the tendinopathic area of the tendon using TEM. This is in line with previous animal studies of Soslowsky and colleagues [33-35], where rats ran with a velocity of 17 m/minute, 5 days/week, 1 hour/day, either uphill or downhill for a period of between 2-16 weeks. In such experiments, a decreased collagen fibre organization and increased numbers of cell nuclei were observed [36,37]. The present TEM analysis did unfortunately not allow for distinguishing between the cell types that were counted, and thus it was not possible to exclude that other cell types than just fibroblasts might have migrated into the t-area of the tendon. The significantly higher mRNA expression of both collagen I and collagen III in the t-area shows a higher collagen synthesis of the tendon. At the same time indicates the higher expression of MMP-2 and MMP-9 in the t-area an increased collagen matrix degradation. Together these findings display a higher collagen turnover in the t-area of the tendon. It has previously been shown that normal tendon tissue express matrix metalloproteinases and that a homeostatic turnover is necessary for tendon regeneration and maintenance [16]. A increased collagen turnover is usually associated with adaptation to exercise [2] or healing of the tendon [38]. It is still puzzling why the increased collagen turnover in the tendons of chronic patients like the present has not resulted in a decrease in symptoms or a healing of the tissue (symptoms range: 0.5-10 years Table 4). However these data are confirmed in other studies also showing increased collagen turnover in tendinopathic tendons [22,24] and in tendon ruptures [39]. Alterations in proteoglycans have previously been associated with tendon pathology [40,41]. Proteoglycans and glycoproteins are essential for the maintenance of homeostasis of the ECM of the tendon and achieve this by regulating the collagen fibril assembly [42]. The upregulation of tenascin-C, and fibronectin is consistent with earlier findings [24,43]. The observed unchanged levels of versican contrasts earlier findings, where a significant down regulation of versican was observed in both tendinopathic and ruptured tendon tissue [40]. This discrepancy might lie in the medical history of the patients. The present patients had a very long history of tendon pain (range: 0.5-10 years). Thus the current biochemical situation in the tissue of these patients may have changed over time. The observed tendency to a decrease of decorin expression in the t-area of the tendon was contrary to our hypothesis. It has been previously shown that a down-regulation of decorin using anti-sense decorin injections improved ligament healing in rabbits [44]. Whether the present finding of a depressed decorin is part of the healing response of the tendons and thus beneficial for the patients is unclear. Additionally the increased expression of fibromodulin in the t-area may partly explain the observation of many thin collagen fibrils since fibromodulin participates in the matrix assembly leading to a delayed fibril formation and formation of thinner fibrils [45,46]. Treatment with injections of growth factors for tendon injuries has received much attention in recent years. Growth factors are polypeptide molecules that are decisive regulation of cell metabolism and cell proliferation and are associated with tendon healing [47-51]. Studies using local administration of bFGF [52,53], HGF [54,55] and IGF [56] have all shown beneficial effects in the healing process of tendon injuries, but not all injuries were tendinopathies though. Exogenous injections of bFGF in human patellar tendons have been shown to increase wound healing both *in vitro *and *in vivo *in patellar tendon models after surgery [52,53], and likewise, collagen type III and cell proliferation was increased after exogenous bFGF injections in patellar tendons after surgery *in vivo *[53]. Recently, our group showed that the cytokine IL-6 could act as a growth factor in tendon tissue [57]. Moreover, local injections of rhIL-6 have been shown to increase collagen synthesis in humans after one hour of exercise in the form of running, in healthy young men [57]. However, the issue as to whether local injections of IL-6 in tendinopathy patients may be beneficial to the healing process of a damaged tendon is still unknown. The pain that tendinopathy patients experience has previously among other been related to Substance-P, a neuro-peptide with various biological functions including pain transmission [58,59]. Since no difference in Substance P expression were observed between the two areas, the present data might indicate that other factors than substance P can be responsible for the pain in tendinopathic tendons. It is however also possible that the expression of Substance-P is increased in the whole tendon and therefore overlooked due to the previously mentioned limitations of the present design. Although the role of inflammation in tendinopathy is one that is often discussed, it has long been known that tendinopathy is primarily a degenerative condition, in which inflammatory cells in or around the lesion are absent. All markers of inflammation that were measured showed no upregulated expression in the t-area of the tendon (Table 3). This underlines that long-term tendinopathy is not primarily an inflammatory process, but rather an ongoing degenerative process. Although inflammation is absent in tendinopathy at this late stage, it does not rule out that an inflammation insult was present at the initiation of the tendinopathic process [60,61]. In fact, various inflammatory mediators like TNF-alpha, IL-6, IL-15, IL-18 have been shown to play a role especially in wound healing after injury [62] and in early stages of tendinopathy [23,63]. However, since inflammation can not be detected in later stages of chronic tendinopathy, the present data may indicate that the use of anti-inflammatory treatments e.g. NSAIDs are not relevant for chronic tendinopathy patients.

**Table 4:** Subjects characteristics

	Age [yr]	Gender M/F	Weight [kg]	Height [cm]	BMI [kg/m^2^]	History of symptoms [Y] (range)
Structural study (*n *= 14)	48 ± 12	11/3	86 ± 17	182 ± 8	26 ± 4	3 ± 2.5 (0.5-10 years)

Biochemical study (*n *= 16)	49 ± 10	10/6	85 ± 18	175 ± 10	28 ± 5	2 ± 1 (0.5-3 years)

Data shown as Mean ± SD

## Conclusion

The present study examined the differences in structural proteins, cellular volume densities and expression levels of various genes involved in regulation of matrix proteins in clinically and ultrasonographiclytendinopathic regions of the human Achilles tendon and healthy regions within the same Achilles tendons. The main findings were differences in the composition of collagen structures with the tendinopathic region containing significantly higher number of small size fibrils (diameter 10-40 nm) compared to the healthy region of the tendon. In addition, the tendinopathic region had a significantly higher volume fraction of cells, compatible with a greater number of cells per unit volume. Furthermore, expression of several genes involved in both collagen synthesis and collagen degradation was significantly up-regulated, an observation that is consistent with an increased local turnover of collagen tissue in the affected tendinopathic area of the tendon. Gene expression was also influenced by the disease as several factors involved in wound healing were expressed at a lower number in the tendinopathic area. Lastly no sign of increased inflammation was found in the diseased region. Taken together these data indicate that local morphological and biochemical differences are present within the tendon during Achilles tendinopathy. These findings may have implications in the choice of treatment for these patients.

## Competing interests

The authors declare that they have no competing interests.

## Authors' contributions

JP carried out the study, made the PCR analyses, made the statistics and drafted the manuscript. UF recruited all patients, took all biopsies. KQ made all the Transmission Electron Microscopy analyses, JOL made all stereology analyses and cell counts, PS and KH supervised the gene expression analyses and PS designed all PCR primers and supervised the statistical analyses. MK and HL participated in designing and coordinating the study and helped to draft the manuscript. All authors read and approved the final manuscript.

## Pre-publication history

The pre-publication history for this paper can be accessed here:

http://www.biomedcentral.com/1471-2474/13/53/prepub
